# Implementation of health promotion programmes in schools: an approach to understand the influence of contextual factors on the process?

**DOI:** 10.1186/s12889-017-5011-3

**Published:** 2018-01-22

**Authors:** Emily Joan Darlington, Nolwenn Violon, Didier Jourdan

**Affiliations:** Laboratoire Acté EA 4281, Chamalières, France

**Keywords:** Implementation, School settings, Realist evaluation, CMO, Contextual factors, Interactions, Programme outcome

## Abstract

**Background:**

Implementing complex and multi-level public health programmes is challenging in school settings. Discrepancies between expected and actual programme outcomes are often reported. Such discrepancies are due to complex interactions between contextual factors. Contextual factors relate to the setting, the community, in which implementation occurs, the stakeholders involved, and the characteristics of the programme itself. This work uses realist evaluation to understand how contextual factors influence the implementation process, to result in variable programme outcomes. This study focuses on identifying contextual factors, pinpointing combinations of contextual factors, and understanding interactions and effects of such factors and combinations on programme outcomes on different levels of the implementation process.

**Methods:**

Schools which had participated in a school-based health promotion programme between 2012 and 2015 were included. Two sets of qualitative data were collected: semi-structured interviews with school staff and programme coordinators; and written documents about the actions implemented in a selection of four schools. Quantitative data included 1553 questionnaires targeting pupils aged 8 to 11 in 14 schools to describe the different school contexts.

**Results:**

The comparison between what was expected from the programme (programme theory) and the outcomes identified in the field data, showed that some of the mechanisms expected to support the implementation of the programme, did not operate as anticipated (e.g. inclusion of training, initiation by decision-maker). Key factors which influenced the implementation process included, amongst other factors, the mode of introduction of the programme, home/school relationship, leadership of the management team, and the level of delegated power. Five types of interactions between contextual factors were put forward: enabling, hindering, neutral, counterbalancing and moderating effects. Recurrent combinations of factors were identified. Implementation was more challenging in vulnerable schools where school climate was poor.

**Conclusion:**

A single programme cannot be suited or introduced in the same manner in every context. However, key recurrent combinations of contextual factors could contribute to the design of implementation patterns, which could provide guidelines and recommendation for grass-root programme implementation.

**Electronic supplementary material:**

The online version of this article (10.1186/s12889-017-5011-3) contains supplementary material, which is available to authorized users.

## Background

When children’s health is considered from a holistic perspective, which includes the physical, emotional and social dimensions [1] of health, the influence of multiple health determinants [2] from children’s life ecosystems becomes evident. A promising strategy to improve children’s health and well-being, is to target such determinants of health [3], with the underlying purpose of addressing and reducing health inequalities. Reducing health inequalities, and promoting children’s health and academic achievement [4] is particularly relevant in school settings: school, as an “ongoing setting where health is created” [5], and a focal point in the community, is a setting of choice to implement health promotion programmes [6] which involve the whole community [7, 8]. However, the path which had been planned in the design of such complex inter-sectoral initiatives is not always followed within the programme, as pointed out by Steward-Brown about the Health Promoting School approach [9]. The nature and expression of programme outcomes are variable. Effects of health promotion programmes are difficult to anticipate and very much dependent on the characteristics of the context of implementation [10]. Discrepancies between expected outcomes and actual programme outcomes are reported [11]. One potential explanation to this, is that programmes and interventions are often blueprinted out-of-context, in total or in part. Expected achievements are often set beforehand, and the potential of the context to deliver them or not is not necessarily taken into account.

In order to scale up the design of strategies and programmes, especially in school settings, it seems relevant to better understand the stakes involved in health promotion programme implementation. This work is a contribution to existing implementation research in the field of health promotion. The purpose of this research is to build knowledge on the processes at play during programme implementation, and the critical conditions and factors which influence such processes, based on existing literature as well as empirical research.

As presented in the literature, assessing and evaluating programme outcomes is quite challenging [12] in the field of health promotion. This is partly due to the very nature of health promotion, which is a process undertaken *with* people and not an end in itself [12]. Rowling and Jeffrey note that programme outcomes are not inferable to programme implementation alone. Results from the implementation of health promotion programmes fall into more than the two obvious categories of either success or failure to achieve pre-defined objectives [12]. Programme outcomes result from complex interactions and are often observable over time [13]. The implementation of a programme is not “a linear trajectory with a beginning and an end” [3], but a non-linear, complex and dynamic process [12] which is sensitive to local context [10]. Exploring how outcomes are generated requires (1) to take into the complex nature of the process from which outcomes result, and (2) to understand how the local context may influence such a process [14].

Implementation research in the field of health promotion [15, 16] has been a major focus over the past years. One of the reasons for this is that researchers have undertaken to build knowledge to enhance programme effectiveness and programme fidelity, especially in schools [17–19]. However, findings put forward that the delivery and sustainability of a programme is difficult to anticipate. When a programme is introduced into local ecology [20], numerous contextual factors [15, 21, 22] influence the implementation process [23]. As Fixsen et al. observe, “like gravity, organizational and external influence variables seem to be omnipresent and influential at all levels of implementation” [15]. Examples of such factors [24] relate to (1) the people [25] involved in programme implementation (e.g. leadership, partnership work, teamwork, motivation, workload [26, 27]), (2) the characteristics of the setting (e.g. organizational capacity [28], turnover [26], team management and management style [29]), (3) the community and its involvement (e.g. policy, funding and support [21], cultural and historical background [30], relationships within the community [31], relationship with school settings, intersectorality [27]), (4) the macro national context (e.g. political and policy organization [32] and funding [17, 33], political and financial commitment and support [27], policy development [33]), to name a few. Additionally, the characteristics of the programme add to this complex situation: e.g. the use of an individual/ecological perspective [34], the duration of the programme [35], the inclusion of training and support [25], the choice of a bottom-up/top-down approach [36], the compatibility with the culture and needs of the setting [27, 29] and adaptability [21], amongst other factors [37]. Moreover, it is often difficult to distinguish which of the intervention components have contributed most to the results observed [38].

### Research focus

This research takes its roots in transformational change [39]. Our standpoint is to consider the dynamic and complex process of interactions [14, 40] between a programme and an existing set of circumstances and conditions. Our point of focus is different from approaches which consider outcomes of this process in terms of individual behaviour change, which is, for example, the case in the mediating variables framework [41]. Outcomes are considered in terms of transformational change across the whole context (e.g. setting, community, stakeholders), and include potential retroaction on the programme. Drawing from a multi-level [42] and complex [43] perspective, outcomes are expected to show on different levels of the local ecology [44]. Outcomes and factors on each level of the local ecology potentially influence other outcomes on other layers of the local ecology [17, 27, 28].

The focus of this research is to understand how interactions between contextual factors influence the implementation process to result in variable programme outcomes. Two leads are undertaken for this research: first, the type of outcomes resulting from programme implementation in a given context [45]. Our approach is indeed firstly to work backwards from outcomes, and to highlight and understand the process which has generated them; secondly to identify the contextual factors and interactions of contextual factors which have influenced the process [14] generating such outcomes and their influence on the process?

### Conceptual framework

Exploring such complex implementation processes in the field of health promotion is challenging. Samdal and Rowling, quoting Deschesnes [27], put forward the difficulty to elaborate “models that can be put into practice in natural contexts” [27]. In recent years, theory-based evaluations [46, 47] have been widely developed and used to address the challenges pertaining to the evaluation of complex health promotion programmes [34, 48]. In theory-based evaluation frameworks, programmes are assumed to operate in non-linear patterns. An embedded implicit or explicit “theory of change” underlies how and why the programme works [49] as anticipated. The development of the theory-based approach has led Pawson and Tilley to include unexpected or negative outcomes as potential results of programme implementation [50]. Realist (or realistic) evaluation focuses on four key questions: *what* works (what kind of programme?), *where* (in which context?), *how* (what are the determining factors and how do they impact the process?) and *for whom* (which stakeholders?). The overall evaluation of a programme can be broken down into 4 steps. (Step 1) Elaboration of the initial programme theory, (Step 2) Data collection, (Step 3) Elaboration of Context-Mechanism-Outcome configuration, (Step 4) Feedback on initial theory.

Our proposal is to use Pawson and Tilley’s framework to explore programme implementation in school settings, without discarding context specifics. Realist evaluation is often used to understand what factors have determined whether the programme was delivered as planned and efficiently. This research is not designed to describe and list a set of favourable or unfavourable conditions in the context, which could have influenced whether the programme was delivered as anticipated. While realist evaluation has indeed much to offer here, its focus is shifted from programme outcomes to the implementation process. Our intention is to analyse and model the interactions occurring between contextual factors, and understand how such interactions have influenced the implementation process. Based on the difficulties experienced with the realist evaluation framework [51, 52] in previous research, terms used in this work are clearly defined as follows. **Expected outcomes** refer to pre-defined outcomes expected to result from the implementation of the programme. As an example, the development of project management skills could be expected to result from the implementation of a training programme in terms of project management. **Programme impact** refers to expected and unexpected outcomes on the whole context, as well as potential retroaction on the programme itself. For example, health capacity building outcomes [53], e.g. organizational changes, changes in leadership or partnership, competency development etc..; and/or changes in the setting [54], e.g. institutional changes, pedagogical and curriculum innovation in school settings; evolution in programme content, and /or achievement of outcomes set beforehand for the intervention. Impact encompasses negative outcomes, e.g. stress, work overload, a project being dropped, dissolution of a partnership. **Contextual factors** influence the implementation process independently or in key combinations [55]. Such combinations are termed ***“Contextual equations”***. They are a snapshot view of the specificities of a context at a given moment in time. Contextual equations are dynamic, and changeable in time. Based on previous empirical research on health promotion programme implementation [24], we argue that recurrences in combinations of contextual factors occur. We term them ***“Typical Contextual Equations (TCE)”***. Typical contextual equations (TCE) could be compared to a setting or community implementation profile. Last but not least, **Mechanisms** account for the interactions between the programme and the context. Mechanisms exist in a loop between an outcome and a contextual factor.

## Methods

### Description of the programme

**Fig. 1:**
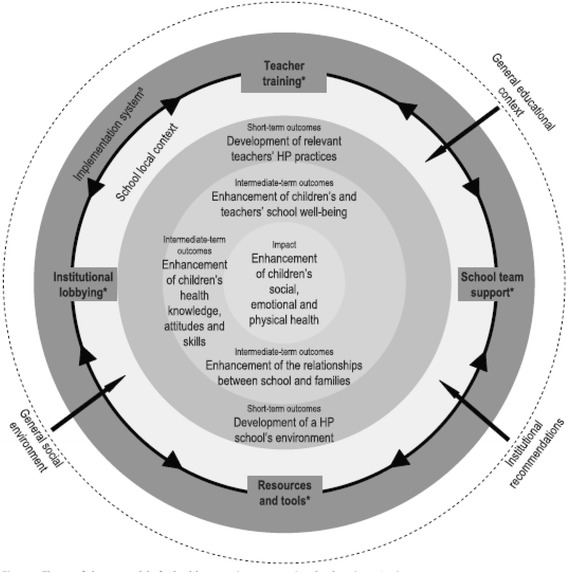
Theory of change used to design the EST programme, from the work by Pommier et al., 2010 [44] & Pommier et al. 2011 [63]

The “Education, Health and Territory” (EST) programme is a school-based health promotion programme. Its design is consistent with the grounding principles of health promotion (reduction of health inequalities, empowerment, focus on socio-economic determinants, holistic and positive approach to health, development of health promotion workforce capacity, community involvement, promotion of intersectorality [56]), as well as principles of the health promoting schools [9] approach (training and support of staff to develop school health policy, focus on school environment and adaptation to local context, community involvement, development of health-related knowledge, skills and competencies [25]). The EST programme, as an offshoot of Pommier and al.’s model [44] for programme design, is underpinned by transformational change [39], as presented in Fig. 1.

Based on relevant international data, the EST programme aims [57] (1) to address health issues in school settings and (2) to provide school staff with the means to develop school health policy, taking into account socio-economic differences within the different school contexts, and to develop sustainable health promotion projects based on the empowerment of local stakeholders; (3) to promote children’s social, emotional and physical health, by promoting their well-being at school, and enhancing their life skills.

An additional file shows the implementation design of the programme in more detail [see Additional file 1].

### Description of the macro-context

The programme was implemented in France between 2012 and 2015. France is a highly centralized country. Yet national education and health policies are adjusted and adapted by decision-makers at the regional level, to fit the needs of local schools. Following the National Health Policy [58], a Health Education Policy is integrated systematically in every “School Project”, which is the strategic document which presents the focus of the yearly school policy plan in a particular school. In French schools, health and citizenship policies aim to promote children’s health and academic achievements. Also, within the school curriculum, health education exists as a transversal entity within existing academic disciplines. Examples of health education learning objectives and content are as follows: to develop social and citizenship competency, to share common values, to promote collective work. Additionally actions and projects implemented in the schools use health promotion and health education strategies and approaches.

### Study design: Using the steps of realist evaluation

Realist evaluation [50, 59] is used to design the study, and collect and analyse the data. As the programme was delivered in the same way in the different schools, we argue that differences between expected outcomes and observable programme impact, are inferable to the differences found in contextual factors and/or combinations of contextual factors in the different school contexts.

### STEP 1: Modelling the programme theory

Programme theory accounts for what kind of achievements were expected from the programme when it was designed by programme leaders. Stakeholders and researchers involved in the programme were consulted to determine which potential contextual factors could influence programme implementation, as implementation had been foreseen. Additionally, results from previous empirical work on similar types of programmes in school settings [24, 44, 60–63], enabled the researcher to design the programme theory.

Expected outcomes on each selected level of implementation were identified as follows:

- *district / community stakeholders*: implementation of the training in schools, and support to school staff;
- *school*: development of a health promoting approach, improvement of the school climate, improvement of home/school relationships;
- *staff*: development of health promotion practices, promotion of partnership work, empowerment;
- *pupils:* enhancement of well-being, promotion of social / physical / emotional health.

Programme theory is presented in Table 1.

**Table 1:** Expected outcomes at different levels of programme implementation, and potential mechanisms and factors involved [57, 63]

Expected outcome at district stakeholders / community level Implementation of the training in schools, and support to school staff	
Potential factors involved	Potential mechanisms
*Institutional support* ^*a*^ Involvement and support of the municipality Involvement, support and ownership of the community Engagement in community networks Stability in the team Stability in administrative structure and procedures	Importance given to HP Accession to the programme *Perception of self-efficacy*^*b*^ to carry out training and support to school staff *Competency development* ^*c*^ *Partnership work* ^*e*^ Reflexivity and sharing of experiences
Expected outcome at school level Development of a health promoting approach, improvement of the school climate, improvement of home / school relationships	
Potential factors involved	Potential mechanisms
*Partnership work* ^*e*^ Implication of families Institutional will Support from local stakeholders Training means and resources Involvement of the community	Shared perception of HP *Integration of HP in the “School Project”* (School yearly policy plan^*d*^) Presence of a leader *Development of collective work* ^*g*^
Expected outcome at school staff level in local schools Development of health promotion practices, promotion of partnership work, empowerment	
Potential factors involved	Potential mechanisms
Size of the implementation area Inclusion of training and support *Collective work* ^*g*^ *Institutional support* ^*a*^ *HP integrated in school project* ^*d*^ Training means and resources Existence of HP approach within the school Perceived needs of children	*Perception of HP* ^h^ *Capacity to integrate HP in their practice* ^f^ Accession to the programme Development of personal skills *Perceived self-efficacy* ^*b*^ Capacity to use resources Motivation and interest Teachers’ empowerment *Leadership* ^1^ Mutualisation.
Expected outcome at children’s / pupil level Enhancement of well-being, promotion of social / physical / emotional health	
Potential factors involved	Potential mechanisms
School characteristics *Staff perception of HP* ^*h*^ *Capacity of staff to integrate HP in their practice* ^*f*^ Duration and content of training (inclusion of support) Improvement of home / school relationship *Collective work* ^*g*^ Development of HP whole school approach Development of HP practice Development of a supportive environment (psycho-social and physical aspects)	Implementation of health education activities Development of personal life skills *Knowledge, competency development* ^*c*^ Critical thinking Involvement of children in health promotion projects

*HP* Health Promotion

Some of the contextual factors presented here are found at different levels of the implementation process in school settings (e.g. institutional support^a^at district and staff levels, perceived self-efficacy^b^, competency development^c^, etc.). Some of the contextual factors are found in the “potential mechanism” category (e.g. integration in school project^d^, partnership work^e^, capacity of staff to integrate health promotion in their practice^f^, development of collective work^g^, perception of health promotion^h^, etc.) as well as the “potential factors” category

Programme theory as presented here consists of a set of four CMO configurations referring to the fours levels of outcomes expected after programme implementation. The four CMO configurations anticipate potential processes and contextual factors, which could theoretically have an impact on the implementation of the programme and influence the expression of subsequent outcomes.

### STEP 2: Quantitative and qualitative data collection

#### Participants

Schools were selected upon their participation in the programme between 2012 and 2015. Overall, three district management teams, 27 schools and 1553 pupils aged 8–11 were included. More focused and thorough data collection involved a selection of four schools from the same educational district. The four schools were labelled as school A, B, C and D.

### Instrumentation

This study was part of a broader research project [64] which used mixed methods [65] to carry out the evaluation of the health promotion programme which was in focus in this work [35]. Overall, 2 sets of qualitative data were collected:

- semi-structured interviews with district pedagogical advisors from two educational districts, three programme coordinators, and a selection of teachers in four schools;
- written documents relating to the programme: minutes of steering meetings (3), minutes of operational team meetings (12), programme presentations (2–6 months apart), intermediate evaluation reports (1), training session evaluation reports (2), results from state of play questionnaire targeting teachers. Documents relating to school actions and practices were also collected. Such documents included minutes of school board (seven in school A, six in school C), school project action sheets (four in school B), teaching session preparation sheet (one in school A), and written documents as well as pictures by pupils.

A validated questionnaire [66] was used to collect **quantitative data**. 1553 paper questionnaires targeting pupils aged 8 to 11 were handed out in 14 schools. Consent for children’s participation was provided by the parents. The topics covered by the set of questions included well-being and school climate. The data extracted from the questionnaires provided information about the different contexts in the different schools.

The analysis undertaken in the study presented is the second phase of the broader research project. As a first step of the broader project, realist evaluation of the programme was carried out [64], in order to screen the large dataset and put forward categories of outcomes and effects which were observable after programme implementation. Contextual factors were also categorized. Results from this work were published in a peer-reviewed journal [64], and provided the basis for the research presented here. However, in this study, the data were approached in a slightly different manner from what was carried out in the overall project. Quantitative data are used as background data, to shed light on the qualitative data in a QUAL/quan embedded [35] perspective.^1^

As detailed above, data relating to outcomes were collected on 3 levels of implementation: Level A - educational district and community stakeholders; Level B – at school level; Level C – at staff level. Outcomes found at pupil level are not considered in this particular work. The data collected on contextual factors have three purposes: to put forward the different categories of factors found in the different school contexts; to characterize the type of influence each factor had on programme implementation; and to elicit potential interactions between factors and the effects of such interactions.

### Data analysis

#### STEP 3: Context-mechanism-outcome (CMO) configurations

##### Screening of the data

The quantitative data are analysed from two perspectives: all the schools selected for the research formed one unit of analysis; a selection of four schools formed another unit of analysis. In this case, the data provide a description of the school context in each school.

Content analysis [67, 68] was used to code the verbatim from interviews and the data extracted from the documents. A “closed approach” was used to categorize the data, using pre-defined categories of expected outcomes and potential contextual factors, which had been identified in the programme theory in step one.

The data were screened twice. The first screening of the data focused on programme impact and the contextual factors and mechanisms that could explain the impact of the programme at the three levels of implementation. The second screening of the data focused on contextual factors, and how such factors influenced the variability in programme impact across the set of schools. This twofold approach provided a more detailed analysis of the interactions at play during the implementation process. In the course of both screenings, mechanisms were deduced when possible.

##### Analysis of the field data

The data were analysed to elaborate two types of CMO configurations: CMOs at the different levels of the implementation process, CMOs for each of the four schools selected for the study. Details of how data analysis was carried out, are presented in Additional file 2.

## Results

### CMO configurations at the three levels of implementation

#### Level A: CMO configurations relating to outcomes at educational district and community stakeholder level

After programme phase 1 (train-the-trainer session), the implementation of the training and the support by district and local stakeholders were found in variable forms and intensity depending on the context. The key contextual factors which influenced the process leading to the variable implementation of the training and the variable support from district staff and community stakeholders were identified and listed as follows (C): the level of implication of the trainers involved, their appreciation of the programme and accession to it, the approval and support from institutional hierarchy, and the development of partnerships. When the programme was given a positive appreciation (C), the support to school level implementation was enhanced (O), and partnerships were developed more easily (O). The positive appreciation of the programme had a positive effect on phase 2 implementation of the training (O) by increasing the implication of trainers (M), which seems to be the underlying mechanism in this configuration. One district team supported teaching staff regularly and followed-up on the programme (O) as coordinators were able to work with schools directly without needing to obtain formal approval from their hierarchy (C). However in the other two districts, no follow-up support was provided (O), as formal approbation from the hierarchy was needed to work with the schools (C). Detailed interactions between contextual factors were put forward and are presented in Table 2.

**Table 2:** Effect of factors on the implementation of the programme at district / community level, and other factors which moderate key factor effect

Key factor: Implication of trainers at district and community level	
Moderator (mod)	Effect on implication
Initiation by decision-maker	Positive effect: facilitated coordination and upscaled available resources. End result was enhanced implication. Negative effect: reluctance of staff to participate in the programme was due to a concern that their practice would be instrumentalized by political decision-makers. Participation was sometimes perceived as being imposed.
Level of delegation from institutional hierarchy	Positive effect: more room for initiative on the part of district teams led to higher implication. The level of delegation was related to the intention of protecting the teams from an “intrusive” programme.
Institutional support from Head of Regional Education Authority	Positive effect: promoted active participation of the health and social district departments. Negative effect: implementation was perceived as following a top-down mode of introduction, and adding constraints. This created reluctance to participate in the programme. Counter-balance: development of relationships between participants counterbalanced the fact that the programme was in most cases imposed by staff at higher decision-making levels. This factors enhanced motivation and implication.
Implementation area	Negative effect: the area referred to electoral districts and not national education districts. This led to tensions and reluctance as all the schools which related to the same school district could not be enrolled.
Name of programme	Negative effect: the name was unclear and determined reluctance to participate in the programme on the part of district teams
Key factor: Appreciation and accession to the programme	
Moderator	Effect on appreciation
Programme content	Positive effect: overall, the programme was appreciated due to concreteness of its content *“for the first time, we went into pragmatic and concrete things”.* Negative effect: suggestions for programme development include: more focus on pragmatic issues, more focus on the needs of the different schools at the very start of the programme. Only one district focused on how staff could engage in the use of the tools introduced during the training. The development of specific content depended on the district staff’s competency (e.g. relaxation sessions). This situation affected school staff’s appreciation of the programme negatively.
Training	Positive effect: inclusion of training in the programme enhanced accession to the programme as staff felt the programme was in line with their expectations.
Key factor: Development of partnerships	
Moderator	Effect on development of partnerships
Institutional support	Positive effect: support from the head of the Regional Education Authority was beneficial to the implementation of the training, and the development of partnership work with the Teacher Training College.
Implication of the Teacher Training College.	Positive effect: implication of the Teacher Training College in train-the trainer sessions had a positive effect on the initiation of partnerships. Moderating factor to implication of the training college: low implication of the Teacher Training Colleges was due to organizational and communication issues between the two institutions.

Key factors were found to influence the results of the implementation process, factors also influenced the type of outcomes which resulted from programme implementation. Other factors were associated to this process. Such factors had different types of moderating effects. Moderators influenced the way in which key factors had an impact on implementation. Moderators may enhance (positively impact) or hinder (negatively impact) the effect of key factors on the implementation of the programme. They may also counter-balance the effect of a key factor, or have no influence at all (neutral effect)

#### Level B CMO configurations relating to the outcomes at school level

At school level, the integration of the programme in the “School Project” (school policy yearly document) (C) had a positive effect on the development of a health promoting approach within the school (O). The underlying mechanism was not identified, but data analysis showed that strong leadership on the part of the management team was beneficial to the institutionalization of the programme in the school policy (Mod.). Another type of impact found on school level was the enrolment of other schools in the programme (O). Schools were recruited to join the programme by some of the schools which had previously participated in the programme, and where a health promoting approach existed and was developed (C). The existence and development of health promoting approaches had a positive spill-over effect on other schools. Staff shared experiences, and colleagues working in other schools heard of the concrete actions which had resulted from their colleagues’ participation in the training. This clearly encouraged the enrolment (M) of new recruits.

#### Level C CMO configurations relating to outcomes at school staff level

- Outcome 1: Participation of school staff in the training

The key contextual factors (C) which influenced the participation of school staff in the training (O) included: how the staff perceived the programme as being adapted to their needs, how the district teams made sense of school staff’s participation in the training, the availability of staff, the training means allocated, the accession of staff to the training, and finally the level of implication of school staff. The fact that the programme included training had a positive effect on the accession of school staff to the programme. The programme was indeed perceived by staff as corresponding to their needs, and this resulted in positive feedback about the programme. The accession of staff to the programme was also influenced by the mode of initiation of the programme. When the programme was initiated by a local decision-maker, this was a key moderator. In cases where the district teams were responsible for the choice of participating schools, staff experienced participation in the training as being imposed upon them, at least in some schools. However, when participation was on a voluntary basis, participation in the programme was not perceived as being imposed by decision-makers and the accession of staff to the programme was enhanced. The name of the programme had a negative effect on the accession of staff to the programme. The inclusion of “Health” and “Territory” in the name of the programme did not seem coherent with what staff expressed of their professional identity. One member of staff stated *“We’re not social workers”*, which sums up the fact that, in some cases, school staff did not feel that the programme was adapted to them. The participation of staff in the training initiated partnerships and in some cases the enrolment of school staff in the school’s “Health Promotion Committee”. More detail about the key factors and how they had an effect on participation of school staff in the training are presented in Table 3.

**Table 3:** Key contextual factors and their effect on participation of school staff in the training

Outcome 1: Participation of staff to training	
Key contextual factor	Effect on participation
Needs assessment and adaptation of the programme	Positive effect: oriented the choice of approach used in the training (e.g. cognitive perspective in line with teachers’ needs) and determined changes and evolution of the training Positive effect 2: the adaptation of the programme to the needs of school staff had a counter-balance effect on the reluctance to participate in the programme, in cases where school staff perceived their participation as being imposed by institutional decision-makers
Importance given by district teams to the programme	Proportional effect: higher priority given to the programme by district staff determined higher participation of school staff. Support from the district management team enabled the training to actually take place in some schools.
Availability of staff	Positive effect: when staff were available they could participate. Negative effect: the means to replace school staff who were participating in the training were not provided. The programme was perceived as an add-on activity in school staff’s busy schedule
Means allocated	Negative effect: insufficient means were allocated which hindered the implementation of the training
Implication of school staff	Positive effect: higher implication of school staff was linked with higher participation of school staff in the training

Results highlighted a number of retroactive effects on the programme: participation of school staff opened new expectations to be taken into account in the design of the training, as well as leads for additions to be made to the training content, e.g. adding focus on the development of psycho-social competencies, including pedagogical resources, tools, and examples of activities, as well as addressing legal and administrative issues.

- Outcome 2: Change in school staff’s health promotion practices.

The training which was included in the programme (C) initiated change in staff’s health promotion (O) practices. Staff’s motivation to implement actions in the classroom was higher (M). Also, staff’s vision of what health promotion practices entailed changed after the training (M). However, in some cases, the training was perceived as lacking concreteness (C), which hindered the development of health promotion practices. In cases where support was provided to school staff to encourage their use of the tools presented in the training (C), staff unsurprisingly used the tools more (O). In terms of the initiation of partnerships, as a mirror of changes in health promotion practices (O), when municipal funding was available (C), punctual actions on health-related themes where carried out (M) which had a positive effect on new partnerships. In schools where the home / school relationship (C) was good, parents participated more in health promoting actions and projects (O). When staff felt that they were lacking support to develop new partnerships (C), it was more difficult for them to identify new partners (O). In this case, staff were left with a feeling of disappointment. Staff’s vision of health changed, as they gave more consideration to the psycho-social and affective dimensions of pupils’ health (O), which, as a ripple effect, had a positive impact on the implementation of activities to promote pupils’ well-being (O). In some cases, the “School project” (yearly policy plan) was modified accordingly (O).

### CMO configurations in the selection of four schools

#### School A: Small rural school (three classes), very high socio-economic status

##### School context / contextual factors

Pupils assessed the climate of the school as “very good” (92%). They felt “very good” at school (81%), had “good relationships” with peers (92,6%) and adults (92,3%). School staff assessed home / school relationship as “very good and based on mutual trust”. The school head showed great leadership and great implication in the programme. The head encouraged his colleagues to participate in the programme. The team was used to working collectively. Cohesion of the team was strong, which facilitated communication within the team. Overall, accession to the programme was high but remained within school staff, particularly teachers. Staff pointed out that lack of available time was a clear barrier. See Table 4 for synthesis of factors.

**Table 4:** Combinations of contextual factors in schools A, B, C and D

School	Contextual factors influencing the implementation process						Outcome
	Leadership of school principal	Team cohesion and collective work	Availability of staff	Home school relationship	Institutional support	Motivation	
A Small rural school Very high SES	*++*	*++*	*-* *Key factor*	*++*	*N/A*	*N/A*	Seldom use of tools Inclusion of action in school project Few partnerships
B Large urban school Medium SES	*++*	*-* *Key factor*	*-* *Key factor*	*++*	*++*	*-* *Key factor*	Few members trained Difficult implementation Programme perceived as add-on and time-consuming Very limited impact
C Large urban school Medium SES Social diversity	*–*	*+/−*	*N/A*	*-* Key factor	–	N/A	Programme not considered a priority No assessment of programme impact
D Medium suburban school Low SES	+/−	+	N/A	- Key factor	+/−	–	Moderate impact and difficult implementation New partnerships

*SES* Socio-economic Status

N/A indicates that no information was provided by the school

##### Programme impact

After their participation in the programme, school staff perceived that the home/school relationship had been improved. There was also an improvement in the school environment. The training changed staff’s attitude towards pupils: staff listened to pupils more, and gave more consideration to pupils’ stress. Staff’s sense of self-efficacy to implement actions was reported as being enhanced. However, the tools introduced during the training were seldom used. Actions which were implemented as a result of the programme focused on well-being, listening skills, mutual help and peer communication. The new knowledge introduced to the pupils related to physical health (body, eating habits and dental hygiene). Actions and projects were replicated and included in the “School Project”. Few partnerships with out-of-school partners were developed.

#### SCHOOL B: Large urban school (ten classes), medium socio-economic status

##### School context/contextual factors

Pupils assessed the school climate as “very good” (87,7%). They felt very “good” at school (82,47%), had good relationships with peers (88,96%) and adults (81,5%). Cohesion of the team was poor. Home / school relationship was assessed “very good” with “great implication of parents”. However, in this school, staff’s motivation to participate in the programme, as well as staff’s accession to the programme were variable in spite of strong support from the institution. The programme was perceived as a time-consuming add-on activity which conflicted with the little time staff considered available. The focus of the programme was unclear. In this school, programme implementation was difficult. Few members of staff were trained, in spite of strong leadership on the part of the school head, and addition of the programme to the school policy plan. Staff had little time to spend on new partnerships. See Table 4.

##### Programme impact

Impact of the programme was perceived as very limited. The actions implemented after participation in the programme focused on enhancing the school climate, promoting autonomy, and organizing recess. Not much consideration was given to innovation as actions were already included in existing pedagogical activities. The subject of well-being and improvement of pupils’ well-being through conflict resolution was included in the “School Project”. The new knowledge introduced to pupils included themes such as way of life, eating habits, and the human body. The tools introduced during the training were seldom used by school staff. School staff pointed out their need for “more hands-on tools”.

#### SCHOOL C: Large urban school (14 classes), medium socio-economic status, social diversity

##### School context/contextual factors

Pupils assessed the school climate as “very good” (85,15%). They felt “very good” at school (83,47%), had “good” relationships with peers (80,24%) and adults (80,29%). In contrast, school staff assessed the school climate as “good”, but reported conflicts and theft. Cohesion of the team was labelled as medium due to important difficulties within the school: home / school relationship was strained, parents were very involved and their expectations from school staff were very high. Other difficulties included trust issues between parents and staff, and cases of psychiatric illness which were reported in some pupils. The programme was not a priority or central in school activities. Support from the institution and leadership on the part of the school head were reported as limited. See Table 4.

##### Programme impact

Staff did not wish to assess programme impact. The actions implemented after participation in the programme focused on themes such as health, conflict management, ill-being, and upgrades to the school-yard. The new knowledge introduced to pupils related to ways of life, eating habits and the human body. One innovation which resulted from the programme was the creation of a pupils’ committee. Partnerships were developed on a one-off delegation basis, when specific issues required to be addressed e.g. security, first aid.... The use of tools was not mentioned by school staff.

#### SCHOOL D: Medium suburban school (six classes), low socio-economic status

##### Contextual equation/contextual factors

The pupils assessed the school climate as being “very good” (85,85%). They felt “very good” at school (79,74%), had good relationships with peers (89,04%) and adults (81,09%). Surprisingly, staff reported the school climate to be “very bad”. They highlighted many conflicts, and cases of verbal and physical violence. Such important difficulties seemed to have impacted programme implementation. Cohesion of the team was good in the face of difficulty, but staff’s motivation to implementation actions and project was low, in spite of their accession to the programme. Institutional support and leadership on the part of the school head were labelled as “medium”. The home / school relationship was not so good. Parents’ implication in school activities was scarce, and staff reported cases of mutual misunderstandings with the parents. Staff pointed out the discrepancies between the values of the programme and the reality they experienced in the school. See Table 4.

##### Programme impact

Impact of the programme was moderate. The actions implemented by school staff as a result from their participation in the programme focused on themes such as way of life, legal regulations, health, recess and “living together”. Actions were either included into existing learning activities, or they were designed as an immediate solution to a significant problem. The use of tools was not mentioned. Training did not enhance staff’s sense of self-efficacy or self-competence. Out-of-schools partners were asked to implement actions in schools on a delegation basis. The new knowledge introduced to pupils included themes such as way of life, eating habits and the human body. Staff expressed expectations for future training sessions on how to manage pupils’ behaviour, and how to implement out-of-school actions on health-related themes. New partnerships were developed with the after-school club, community centre, however on a delegation basis.

In each school, key factors were identified as clearly determining what type of impact resulted from the implementation of the programme. Different combinations of key factors led to different types and expression of the impact of the programme as presented in Table 4. For example, in school A, strong leadership on the part of the school head was associated with strong cohesion in the staff team, a habit to work collectively in the team, good home / school relationship but little time available for staff to implement projects. This situation resulted in the fact that staff seldom used the tools provided by the training. Also few partnerships were developed in spite of the inclusion of actions in the school policy. In school D, the home / school relationship which had been labelled as bad, and staff’s motivation to implement actions as being low, both overpowered the strong leadership demonstrated by the school head, strong team cohesion, and support from the institution, which could have had a positive effect on the impact of the programme. Implementation of the programme in this school was in fact difficult, and the programme had very moderate impact.

### STEP 4: Feedback on initial theory

This step involved consultation of stakeholders via group interviews and review of the research work.

#### Outcomes, mechanisms and contextual factors

Our work put forward the substantial differences between the outcomes expected in the programme theory and those which were extracted from the field data. At district / community stakeholder level, the implementation of the training was delivered in a variable manner, and did not necessarily correspond to what had been anticipated in the programme design. This was also the case of the support provided to school staff. This situation had an impact on the development of a health promotion approach in the schools. The level of participation of school staff was not as it had been anticipated either. However, school staff’s participation in the training changed their vision of health, and their attitude towards health-related projects clearly shifted. The activities implemented as a result of the programme were focused on a more holistic perspective of health and well-being. Also, some form of enhancement of the home-school relationship was reported but no clear relation with the programme could be made based on the data.

Differences between the expected outcomes, set beforehand by project leaders, and actual outputs of the programme in the different contexts were made evident on different levels of implementation. As an example, accession to and participation in the programme were lower than expected. Accession to the programme and participation were considered to be expected outcomes in this programme. One of the reasons for training to have been integrated in the programme design was precisely to improve accession and participation*.* Also*,* the support from intermediate implementation levels was variable in its form and intensity, while the programme integrated support as an intermediate, or first phase, outcome. Other types of differences from what had been integrated in the design of the programme, and forms of variability in the outcomes were observed: variable usage of the tools proposed, different modes of implementation, different types of collaborations, and different types of partners involved. In terms of capacity building [69] at staff level, outputs ranged from none to the enhancement of the sense of legitimacy and self-competence, the development of new competencies, knowledge and skills, opportunities for curriculum development, the opening of new leads for reflection, enhancement of motivation, the strengthening of their sense of legitimacy and their convictions. It has to be noted, that, unsurprisingly, in more vulnerable schools, where school climate was poor, programme implementation faced even greater difficulties and resulting outputs showed even greater differences.

#### Combinations and interactions between factors

Results from the various studies highlight that contextual factors influence different aspects of the implementation process**:**

- whether the programme generates expected outcomes or outputs at the different levels of implementation;
- how the programme operates at the different levels of implementation;
- how other factors influence the process, i.e. one factor may moderate, potentiate or counter-balance the effect of another factor on the process;
- how the programme evolves and is sustained, as some factors retroact on the programme.

Five types of effects were identified: (1) hindering, i.e. opposing the process as it was expected to happen; (2) moderating, i.e. influencing another factor which is either an enabling or hindering factor to the process as it was expected to happen; (3) counter-balancing, i.e. cancelling out, the effect of a hindering factor on the process as it was expected to happen (4) enabling, i.e. supporting the process as it was anticipated to happen, (5) neutral, i.e. showing no effect on the process, or on other factors.

Some factors acted on more than one level of implementation, e.g. the name of the programme hindered participation at district level, which impacted implementation at school level, and staff participation in the training. A cascade of effects occurred between different levels of implementation, e.g. in some cases, when the programme was initiated by a regional decision-maker, district staff were reluctant to participate in the programme. This situation impacted whether the decision-makers were willing to delegate decision-making power to the district staff. This further impacted support to school staff, as higher delegation led to higher support thus stronger implementation at school level [6, 21, 34].

Recurrent combinations were searched for at different levels of implementation. Such recurrent combinations were termed *“Typical Contextual Equations” (TCE)*:

##### TCE at district level

Three factors, which were key determiners to the implementation process, were found to be combined repeatedly at district level**:** support from the Regional Education Authority [27]; support from the institutional hierarchy [35]; and implication of district and community stakeholders [32, 34, 70].

##### TCE at school level

Recurrent combinations of factors were not identified at this level. Six contextual factors were taken into account, comparisons were made between the schools; however but no TCE emerged (See Table 4).

##### TCE at staff level

Four factors were found to be combined recurrently: encouragement and support to staff by district team [35, 44]; availability of staff [27]; means allocated [27]; and implication of staff [25].

Overall, this programme did not generate equivalent outcomes in every school although it had been designed to work and adapt to any context. It seems in more vulnerable schools, where school climate [44] is poorer, staff faced even greater difficulties during programme implementation. Altogether, no single theory could be drawn from the results, as combinations of determining factors were highly specific and variable.

## Discussion

Some of the factors and mechanisms identified in the programme theory were not activated as anticipated, for example accession [71] to the programme did not necessarily determine action. Nor did the inclusion of training, which had been thoroughly documented as an enabler of implementation [15, 21, 71]. The fact that the programme was initiated by a community decision-maker [21, 70, 72] did not strengthen the seemingly weak ties between the schools and the community. The top-down [36, 73] implementation mode impacted the implementation of the programme greatly. This conclusion from the results was unexpected as the inclusion of training [25] and support from the hierarchy [28] and the community [21, 27] were documented as levers to successful programme implementation in other works. From an operational point of view, this result questions the hierarchical relationships [57] between management teams and local school staff. Programmes cannot be implemented in French schools without the approbation of decision-makers at the district level. However depending on the way the programme was brought to the school by district level staff, the results of this process were different. The relationship between school staff and district level decision-makers of school management influenced the opportunities for school staff to show ownership and develop action. This point emphasizes the need to give attention to programme initiation modes, and emphasizes the importance of negotiated planning [21, 26, 27]. Leadership and support from the school head, which were expected to enhance the accession of school staff to the programme, and the implementation of activities by school staff [17, 71], was not a lever as anticipated. The quality of home / school relationship [57] seemed to overpower the beneficial effect of leadership on the part of the school head in cases where relationships between parents and staff were very poor. Positive relationships and partnerships had already been put forward as a key factor by Mcisaac et al. [74]. School climate [44] was also an influential factor. A potential explanation for this, is that staff who are subjected to a poorer school climate and faced with the serious difficulties of their pupils will increase their chances of experiencing professional exhaustion or burnout. Han & Weiss point out that school staff who experience full professional burnout or degrees of professional exhaustion would not devote any extra time for school projects or actions [71].

Some of the features in the programme influenced the process of implementation, in line with what other authors have pointed out [15, 21, 71]. The content of the training was perceived as not being pragmatic and operational enough. Also, the support provided to develop partnerships was reported as insufficient or lacking. One factor that was not anticipated was the name of the programme, which generated some reluctance to participate. Staff did not make sense of the name of the programme, which was unclear to them and inconsistent with their professional identity [57]. When staff perceived the programme as meeting their needs [21], and as coherent with existing practice [25, 28], their implication was higher. Implication was greatly hindered in the opposite scenario. These findings are consistent with conclusions from other studies [26, 27]. School staff did point out that in cases where the values conveyed through the programme were inconsistent with the reality of school life, even though school staff would adhere to such values, programme implementation was difficult.

The way in which the programme was brought to the schools played an important role in how further implementation took place. Different types of initiation modes were pinpointed: institutional initiation had a negative impact on further implementation, when the relationship between school staff and decision-maker was poor; but had a positive influence on school staff when it was perceived as a credential to what they were already doing [35, 75]. Overall, top-down [36, 73] initiation modes affected further implementation of the programme in a negative way. It would appear that the challenge for school staff was to identify areas, in which they could show ownership, and appropriation. Basically, when they felt that they were in control of successful project outcomes, initiation mode did not have as much of an impact.

### Realist evaluation with a twist

Authors have pointed out that one of the issues pertaining [51, 52, 76] to the use of realist evaluation is how to define the three key terms: contextual factors, mechanisms and outcomes. In addition to this, deciding which category to assign to an item of data is arduous. During this study, it became apparent that working backwards from outcomes was a way to ensure, as far as possible, that what was explored related to the implementation of the programme. The use of this form of configuration enabled the researcher to narrow down the scope of collection, and ensured more consistency and stability in realist evaluation definitions. The same framework was used in another study belonging to a broader research project [37]. As the need to work with a stable set of definitions was underlined throughout the research project, the researcher used the causal loops [77] framework to model interactions and identify how contextual elements and outputs are linked within the whole dynamics of the process of programme implementation as presented in Darlington et al. (2017) [24]. However, we wish to note that any configuration derived from this framework strongly relies on data collection, and it can be argued that contextual equations only partially account for what happened during the implementation process. The degree of complexity in the interactions considered may have caused confusion, which is also one of the limits of this work. As a result of this analysis, CMO configurations were found to be intertwined between different levels of implementation. This emphasizes the need to define what level of implementation is considered when programme outcomes are explored. Any given item may indeed be categorized as an outcome, a factor or a mechanism, depending on the level of programme implementation which is in focus during data analysis.

### Implementation and intervention design

Even in cases when reported levers [15, 17, 21, 27, 57] are activated (e.g. inclusion of training, support from the hierarchy, professionals’ motivation), programme impact is not as expected. Yet, the potential of programmes is not in question here. It is the design of programmes and most of all the aim of the implementation process which requires reflection. Rather than “standardized” interventions replicable across sites, it is the function of the intervention, that is standardized, to enable its form to vary across contexts [48]. Programme implementation can be used to create and develop life ecosystems which promote health for all and reduce health inequalities. The aim of such implementation processes is to (1) enhance the capacity of both settings and communities to promote people’s health, and (2) develop health capacity building. The programme becomes a means to achieve different steps in this process. Adequate achievements and goals can be set in coherence with the potential of the context. This requires to take into account existing enabling or hindering factors and the relationship between them. Selected programme features have to be included in the design of the programme based on the assessment of context specifics. This suggests that a single programme cannot be transferred to all contexts, as “one size does not fit all”. The way a programme is introduced should also be tailored to contextual specificities.

### Strengths and limitations

This work proposes to use the realist evaluation framework in a different way from the method published in the initial book by Pawson and Tilley [59]. Our framework uses a dynamic and complex model of CMO configurations. In the presented work, the use of this model enabled the researcher to put forward that CMO configurations are in fact dynamic constructions which need to be anchored and stabilized by using a starting point. Also, the point of focus has to be identified beforehand, e.g. the level of programme implementation which is explored. To our knowledge, this method of using Pawson and Tilley’s framework had not yet been tested or published.

In terms of the limitations of this work, the following issues stand out. Data collection was not comprehensive. Some documents were not acquired, as schools did not wish to share them with the researchers (e.g. some school projects), or no record was kept. Programme impact was not entirely accessible, and important data may have been overlooked or discarded. The process of categorization was strenuous and difficult. Outcomes were sometimes difficult to infer to school staff’s participation in the programme and could have been the consequence of another phenomenon: e.g. motivation and drive to implement actions could have existed in the schools before the programme was introduced. The vast area covered by the collection of the data added complexity to the research process. Additionally, as paper format was used to collect some of the data, in some of the schools few data were collected. The set of data was found to be inadequate when it came to asserting the validity of the Typical Contextual Equations approach. In addition, the mechanisms involved in CMO configurations were difficult to identify. More work could have been done to conceptualize mechanisms [78], and collect evidence of mechanisms in the data.

### Implications for future development of public health programme implementation

Actions which are rooted in the community [79] and in children’s living environments show greater potential to reduce health inequalities [80]. Schools, are among the building blocks of community grounds and are also key components of children’s living environment [8]. Schools offer great resources and opportunities for the implementation of health promotion programmes. However, programme implementation is particularly challenging [4, 5, 17] in school settings, due to organizational issues [74], among other factors. The strong and specific professional identity of school staff clearly guides what type of action is undertaken [25]. The potential difficulties, opportunities, levers and barriers as regards health promotion programmes in school settings, which we attempted to present in this work, need to be anticipated and addressed before a new programme is introduced. In schools, staff, out-of-school professionals from the education, social and health sectors, as well as stakeholders and parents are brought together, and encouraged to work collectively on projects. This process introduces complexity from the start of programme implementation and even before a programme is introduced. Programme implementation needs to be tailored to the expectations of stakeholders, adapted to their needs, and the resources which are available, as well as flexible enough to overcome potential difficulties. We suggest that an extensive assessment of the specificities of the school and its surrounding environment is required prior to any form of programme implementation. More attention is needed in disadvantaged school contexts, as it seems that it is more difficult for school staff to embrace a global approach towards health. In such schools, ecological interventions are most necessary to promote and sustain pupils’ health [81].

## Conclusion

The use of the realist evaluation contributed to unpacking the process of programme implementation in school settings. A few adaptations were made to the framework in the course of the research. Contextual factors interact in highly complex loops. Effects of such interactions are of five different types. This work is a contribution to implementation research in school settings and in the field of health promotion. Further research is needed to identify contextual equations in other settings and communities, and to compare combinations of contextual factors, the effects of interactions between factors as well as to identify the nature of key factors. The transferability of the typical contextual equation approach requires to be tested and confronted to other field experiences. Further research is needed on how different types of programmes operate in different types of contexts. This would provide useful guidelines and recommendations for health promotion programme designs.

## Additional files

Additional file 1: Programme implementation design. Detailed stages of the implementation of the programme. (DOCX 19 kb)

Additional file 2: Analysis of field data. Detailed stages of data analysis. (DOCX 16 kb)

## Abbreviations

C: Context
CMO: CMO configurations derived from the data
CMO: Context-Mechanism-Outcome
HP: Health promotion
M: Mechanism
Mod: Moderator
N/A: Not Applicable
O: Outcome
SES: Socio-Economic Status
ThCMO: Theoretical CMO configuration from the initial programme theory

## Footnotes

1 In the QUAL/quan approach, the set of quantitative data has a supportive and secondary role to provide additional information to the main set of data, in this case the set of qualitative data. The broader research project involved statistical analysis of the quantitative data which shall not be presented here.
