# Supplementary material for: Implementation of health promotion programmes in schools: an approach to understand the influence of contextual factors on the process?
Source: BMC Public Health. 2018 Jan 22;18:163. doi: 10.1186/s12889-017-5011-3 (PMC5776776; doi:10.1186/s12889-017-5011-3)
Supplement: Supplementary file 1 — Programme implementation design. Detailed stages of the implementation of the programme. (DOCX 19 kb) [file 12889_2017_5011_MOESM1_ESM.docx]

Programme implementation design

The programme was pre-tested and implemented in 6 regions in France. Organizational bodies involved included:

- - a steering committee: policy decision-makers, researchers and a regional authority management team;
  - an operational team: the regional education authority and local management teams, trainers and municipal representatives, in charge of reporting and following-up on the project;
  - a programme coordinator in charge of liaising between the steering committees, the operational teams and the local teams, and provided feedback on questionnaires filled out by school staff and pupils regularly.

In stage 1, the district support service joined community trainers in train-the-trainer sessions, to prepare the training of school staff organized in stage 2. Training sessions used participatory pedagogy to provide professionals with opportunities to connect the project with their own experiences, practice and knowledge. In addition to stage 2 training, support and resources were on offer to promote the development of school-based interventions. (See Table 1 and Violon et al., 2016 for more detail on training content^58^).

**Table 1: Implementation Stages**

| **IMPLEMENTATION STAGES** |
| --- |
| **STAGE 1** |
| Train the trainer sessions ( 3x 2 days) |
| **Trainers:** researchers, regional education authority technical advisor, expert trainer from the network of teacher colleges, head teachers, territorial officers  **Trainees:** local community stakeholders, e.g. district management team, Local Education Authority medical and social workers, head teachers, association representatives, municipal representatives, teacher trainers  **Objectives**:   - to get to know the programme better - to acquire knowledge, skills and competencies to implement a health promoting approach - to develop sufficient training skills to train and support local staff |
| **STAGE 2** |
| Training of local stakeholders (2 day-conference + in-service training) |
| **Trainers:** national education staff: pedagogical advisors, national education inspectors, district nurses, district doctor, head teachers  **Trainees:** local stakeholders including school teachers  **Objectives:**   - to introduce and present the programme, its underlying principles and values - to reflect on the physical and social environment of school - to focus on health and citizenship education, social climate - to set up strategies and put forward leads for future interventions |
| Support to local school staff by district management teams |
| Provision of tools and resources for action, which included activity worksheets, health education children’s books, and an online platform (database and tool for dialogue and communication) |
