# Supplementary material for: Implementation of health promotion programmes in schools: an approach to understand the influence of contextual factors on the process?
Source: BMC Public Health. 2018 Jan 22;18:163. doi: 10.1186/s12889-017-5011-3 (PMC5776776; doi:10.1186/s12889-017-5011-3)
Supplement: Supplementary file 2 — Analysis of field data. Detailed stages of data analysis. (DOCX 16 kb) [file 12889_2017_5011_MOESM2_ESM.docx]

Analysis of field data

Elaboration of Context-Mechanism-Outcomes configurations at different levels of implementation process

A first chart was made with three columns: *contextual factors*, which were then categorized into programme related-factors, school-related factors, and staff-related factors; *mechanism / nature of the relationship,* and *programme outcomes / impact.* ~~.~~ The researcher used the categories of factors identified in the programme theory (Step 1) as leads to categorize the data. The researcher kept an open mind as to what type of outcomes could have resulted from programme implementation. Items categorized as outcomes consisted of the results of the programme, as expressed in the interviews and the documents. The items extracted from the data were not assigned to either the context, the mechanism or the outcome category on the basis of which category each item belonged to in the programme theory. The following sentence was used by the researcher to categorize the data and ensure that definitions remained stable throughout the whole study.

*“Any given element of the context (contextual factor) is a facilitator or barrier (nature of effect) and has an end result (outcome) by doing such and such (mechanism)”.*

For example, institutional support (contextual factor) had a beneficial effect (nature of effect: facilitator) on the implication of teachers in the training (outcome) by enhancing their sense of legitimacy (mechanism). This framework was also applied systematically during data analysis. As the researcher was filling the chart, it became apparent that within the three columns, sub-CMO configurations could be found. A given contextual factor influenced more than one outcome; an outcome could also be a contextual factor which influenced another level of implementation in a cascade effect. At this stage, the researcher chose to design another chart listing the different outcomes and linking them with contextual factors and mechanisms. The same process was undertaken in a third chart, starting from contextual factors, then moving onto the type of effect such factors had on programme outcomes and other potential factors which could have moderated this process. The same wording of items was used to enable the researcher to cross-analyze the charts and put forward interactions between items which appeared more than once in the charts. As a last step, the researcher categorized the data again, this time using the expected outcomes identified in the programme theory. This step ensured stability of the model of interactions, as well as stability in the links between the different CMO configurations which were modelled from the data. CMO configurations were modelled and are presented in the results section. Bracketed letters refer to Context (C), Mechanisms (M), and Outcomes of programme implementation (O). A new category emerged during analysis: it was termed Moderator (Mod.). Moderators were contextual factors which influenced the way in which another contextual factor affected the implementation process. As an example, when the relationship between school staff and the management team was strained (C), if the programme received institutional support (Mod), school staff perceived participation to the training as being imposed upon them (M), which, as a ripple effect, had a negative impact on the creation of health promotion projects by school staff (O).

Elaboration of Context-Mechanism-Outcomes configurations for each of the four schools selected for the study

The dataset was screened to collect data relating to the selection of four schools, and elaborate CMO configurations at school level. The quantitative data was used to describe the school context for each school, and to identify contextual factors which influenced programme impact. Additional information about contextual factors, and programme impact were collected from the interviews which had been carried out with the staff in each of the schools. The framework sentence presented above was used to divide the data into two categories: context and impact. The same items of contextual factors were considered in all the schools, to make comparisons between schools possible, and to identify potential key combinations of factors. The same principle applied to the items relating to programme impact, which were comparable between the schools. A chart was made to compare the types of combinations of contextual factors which could be found in the four schools (See Table 4).
